# The British Hospitals Association

**Published:** 1921-05-14

**Authors:** 


### The British Hospitals Association.

A MEETING of the West Midlands Regional Committee was held at the General Hospital, Birmingham, on April 28, for the purpose of considering evidence to be presented before Lord Cave's Parliamentary Committee of Inquiry. Mr. C. H. Saunders, of the General Hospital, Birmingham, took the chair, and a draft report, in reply to the questionnaire issued by Lord Cave's Committee, was freely discussed. Strong opposition was expressed to any scheme of maintenance charges to in-patients. Mr. Thomas Ratcliff was elected a representative of the Committee to give evidence on their behalf, and a vote of thanks was passed to Mr. W. H. Harper, House Governor and Secretary of the General Hospital, Wolverhampton, for the trouble he has taken in compiling the information placed before the meeting.

